# Supplementary material for: Prenatal Detection of a FOXF1 Deletion in a Fetus with ACDMPV and Hydronephrosis
Source: Genes (Basel). 2023 Feb 23;14(3):563. doi: 10.3390/genes14030563 (PMC10048226; doi:10.3390/genes14030563)
Supplement: Supplementary file 1 [file genes-14-00563-s001.zip › genes-2187531-supplementary/Supplementary files/Table S1_Jan12.2023.pdf]

Table S1. List of identified informative single-nucleotide variants within the copy-number variant deletion region, showing that it involves maternal chromosome 16.

Position [hg38]	Father	Mother	Child	Parent of origin of CNV deletion
chr16:86 411 378	Total count: 32<br>A: 20 (63%, 13+, 7-)<br>C: 12 (38%, 6+, 6-)<br>G: 0<br>T: 0	Total count: 30<br>A: 0<br>C: 30 (100%, 15+, 15-)<br>G: 0<br>T: 0	Total count: 14<br>A: 14 (100%, 9+, 5-)<br>C: 0<br>G: 0<br>T: 0	Mother
chr16:86 432 741	Total count: 33<br>A: 0<br>C: 15 (45%, 7+, 8-)<br>G: 18 (55%, 8+, 10-)<br>T: 0	Total count: 33<br>A: 0<br>C: 0<br>G: 33 (100%, 22+, 11-)<br>T: 0	Total count: 11<br>A: 0<br>C: 11 (100%, 7+, 4-)<br>G: 0<br>T: 0	Mother
chr16:86 433 639	Total count: 21<br>A: 13 (62%, 9+, 4-)<br>C: 0<br>G: 8 (38%, 4+, 4-)<br>T: 0	Total count: 26<br>A: 0<br>C: 0<br>G: 26 (100%, 11+, 15-)<br>T: 0	Total count: 13<br>A: 13 (100%, 6+, 7-)<br>C: 0<br>G: 0<br>T: 0	Mother
chr16:86 519 037	Total count: 38<br>A: 18 (47%, 9+, 9-)<br>C: 0<br>G: 20 (53%, 12+, 8-)<br>T: 0	Total count: 30<br>A: 0<br>C: 0<br>G: 30 (100%, 10+, 20-)<br>T: 0	Total count: 18<br>A: 1 (100%, 11+, 7-)<br>C: 0<br>G: 0<br>T : 0	Mother
chr16:86 541 032	Total count: 34<br>A: 14 (41%, 6+, 8-)<br>C: 0<br>G: 20 (59%, 8+, 12-)<br>T : 0	Total count: 30<br>A: 0<br>C: 0<br>G: 30 (100%, 12+, 18-)<br>T : 0	Total count: 20<br>A: 20 (100%, 8+, 12-)<br>C: 0<br>G: 0<br>T: 0	Mother

chr16:86 550 168	Total count: 43<br>A: 26 (60%, 10+, 16-)<br>C: 0<br>G: 0<br>T: 17 (40%, 8+, 9-)	Total count: 34<br>A: 0<br>C: 0<br>G: 0<br>T: 34 (100%, 15+, 19-)	Total count: 18<br>A: 18 (100%, 6+, 12-)<br>C: 0<br>G: 0<br>T: 0	Mother
chr16:86 571 213	Total count: 29<br>A: 0<br>C: 14 (48%, 5+, 9-)<br>G: 0<br>T: 15 (52%, 6+, 9-)	Total count: 31<br>A: 0<br>C: 31 (100%, 17+, 14-)<br>G: 0<br>T: 0	Total count: 23<br>A: 0<br>C: 0<br>G: 0<br>T: 23 (100%, 11+, 12-)	Mother
chr16:86 572 446	Total count: 25<br>A: 0<br>C: 13 (52%, 7+, 6-)<br>G: 12 (48%, 7+, 5-)<br>T: 0	Total count: 21<br>A: 0<br>C: 0<br>G: 21 (100%, 12+, 9-)<br>T: 0	Total count: 23<br>A: 0<br>C: 23 (100%, 14+, 9-)<br>G: 0<br>T: 0	Mother
chr16:86 574 671	Total count: 41<br>A: 0<br>C: 0<br>G: 18 (44%, 8+, 10-)<br>T: 23 (56%, 11+, 12-)	Total count: 36<br>A: 0<br>C: 0<br>G: 1 (3%, 1+, 0-)<br>T: 35 (97%, 15+, 20-)	Total count: 11<br>A: 0<br>C: 0<br>G: 11 (100%, 6+, 5-)<br>T : 0	Mother
chr16:86 575 180	Total count: 30<br>A: 0<br>C: 10 (33%, 8+, 2-)<br>G: 0<br>T: 20 (67%, 5+, 15-)	Total count: 40<br>A: 0<br>C : 0<br>G: 0<br>T: 40 (100%, 17+, 23-)	Total count: 21<br>A: 0<br>C: 21 (100%, 9+, 12-)<br>G: 0<br>T: 0	Mother
chr16:86 575 293	Total count: 29<br>A: 16 (55%, 7+, 9-)<br>C: 13 (45%, 6+, 7-)<br>G: 0<br>T: 0	Total count: 33<br>A: 33 (100%, 16+, 17-)<br>C: 0<br>G: 0<br>T : 0	Total count: 12<br>A: 0<br>C: 12 (100%, 5+, 7-)<br>G: 0<br>T: 0	Mother
chr16:86 575 696	Total count: 46<br>A: 0<br>C: 26 (57%, 13+, 13-)<br>G: 0	Total count: 37<br>A: 0<br>C: 37 (100%, 23+, 14-)<br>G: 0	Total count: 18<br>A : 0<br>C: 1 (6%, 1+, 0-)<br>G: 0	Mother

	T: 20 (43%, 12+, 8-)	T: 0	T: 17 (94%, 6+, 11-)	
chr16:86 575 760	Total count: 54<br>A: 0<br>C: 30 (56%, 14+, 16-)<br>G: 0<br>T: 24 (44%, 12+, 12-)	Total count: 40<br>A: 0<br>C: 40 (100%, 24+, 16-)<br>G: 0<br>T: 0	Total count: 20<br>A: 0<br>C: 1 (5%, 1+, 0-)<br>G: 0<br>T: 19 (95%, 9+, 10-)	Mother
chr16:86 577 785	Total count: 23<br>A : 0<br>C : 0<br>G : 13 (57%, 9+, 4-)<br>T : 10 (43%, 6+, 4-)	Total count: 12<br>A : 0<br>C : 0<br>G : 0<br>T : 12 (100%, 4+, 8-)	Total count: 15<br>A : 0<br>C : 0<br>G : 15 (100%, 4+, 11-)<br>T : 0	Mother
chr16:86 609 943	Total count: 43<br>A: 24 (56%, 15+, 9-)<br>C: 0<br>G: 19 (44%, 10+, 9-)<br>T: 0	Total count: 23<br>A: 0<br>C: 0<br>G: 23 (100%, 9+, 14-)<br>T: 0	Total count: 21<br>A: 21 (100%, 11+, 10-)<br>C: 0<br>G: 0<br>T: 0	Mother

Total count - total number of reads at particular position. The number of reads for each nucleotide at a given position is shown for family members. “CNV” – copy-number variant; “%”– percentage of reads for particular nucleotide; “+”– number of reads for particular nucleotide, plus strand; “-”– number of reads for particular nucleotide, minus strand.
