# Supplementary figures and images for: Prenatal Detection of a FOXF1 Deletion in a Fetus with ACDMPV and Hydronephrosis
Source: Genes (Basel). 2023 Feb 23;14(3):563. doi: 10.3390/genes14030563 (PMC10048226; doi:10.3390/genes14030563)

A

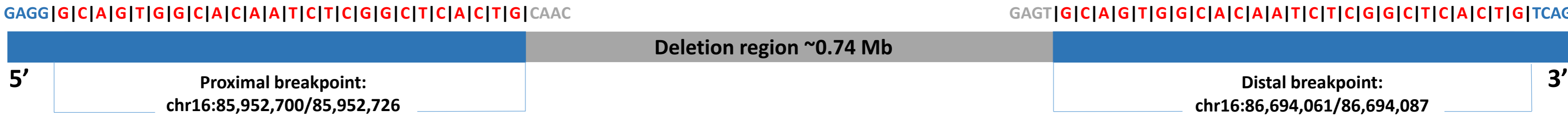

B

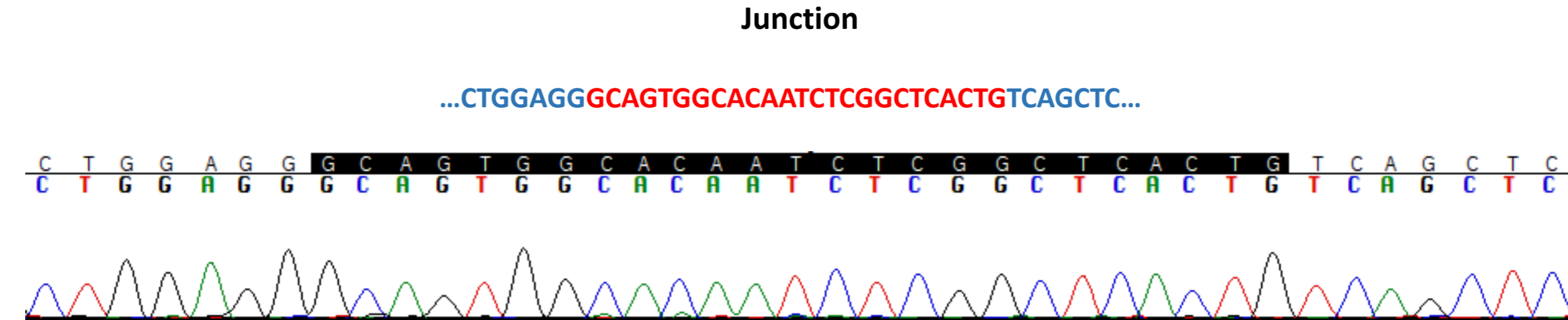

Supplement: Supplementary file 1 [file genes-14-00563-s001.zip › genes-2187531-supplementary/Supplementary files/Figure S1_Jan12.2023.pdf]
